# Supplementary material for: Enhancement of Dendrobine Production by CRISPR/Act3.0-Mediated Transcriptional Activation of Multiple Endogenous Genes in Dendrobium Plants
Source: Int J Mol Sci. 2025 Feb 11;26(4):1487. doi: 10.3390/ijms26041487 (PMC11855359; doi:10.3390/ijms26041487)
Supplement: Supplementary file 1 [file ijms-26-01487-s001.zip › ijms-3432578-Supplementary Materials.pdf]

Supporting Information

Enhancement of Dendrobine Production by CRISPR/Act3.0-Mediated Transcriptional Activation of Multiple Endogenous Genes in *Dendrobium* Plants

Meili Zhao#[1,2], Zhenyu Yang#[1,3], Jian Li[1,2], Feng Ming[3], Demin Kong[1,2], Haifeng Xu[1,2], Yu Wang[1,2], Peng Chen[1,2], Xiaojuan Duan[1,2], Meina Wang*[1,2], Zhicai Wang*[1,2]

1 Shenzhen Key Laboratory for Orchid Conservation and Utilization, the National Orchid Conservation Center of China and the Orchid Conservation & Research Center of Shenzhen, Shenzhen 518114, China

2 Key Laboratory of National Forestry and Grassland Administration for Orchid Conservation and Utilization, the National Orchid Conservation Center of China and the Orchid Conservation & Research Center of Shenzhen, Shenzhen 518114, China

3 Shanghai Key Laboratory of Plant Molecular Sciences, College of Life Sciences, Shanghai Normal University, Shanghai 200234, China

#The authors contributed equally to this work

***Corresponding Authors**

Email: wangmn@sztcg.com (M.W.); wangzc@cnocc.cn (Z.W.)

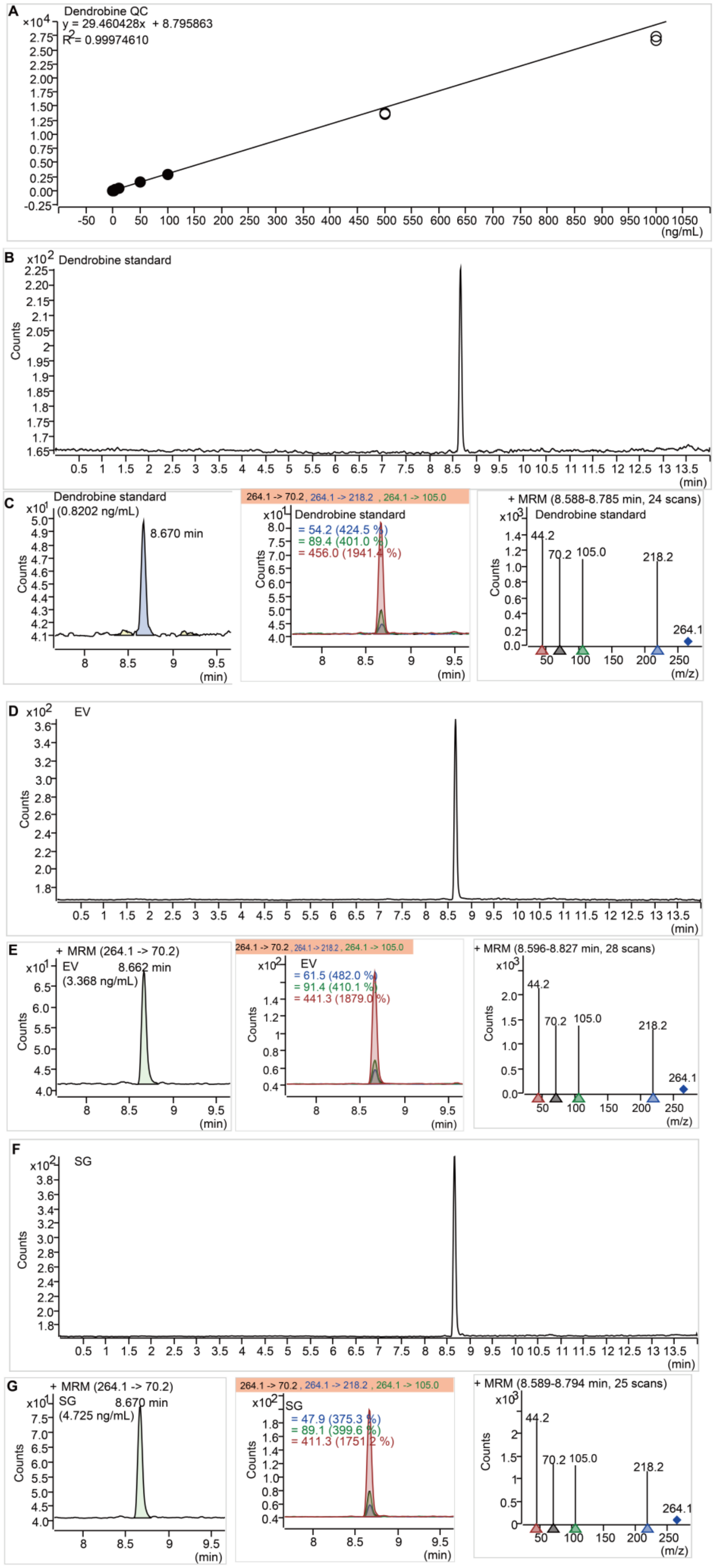

Figure S1. Validation of the LC/MS quantification method using SG-multigene transgenic *D. catenatum* as an example. (A) Calibration curve for a dendrobine standard. (B) Liquid chromatogram of the dendrobine standard. (C) Qualitative and quantitative of the

dendrobine standard by LC/MS; the retention time for the dendrobine standard is 8.67 min. (D) Liquid chromatogram of dendrobine in EV transgenic *D. catenatum*. (E) Qualitative and quantitative analysis of dendrobine by LC/MS in EV transgenic *D. catenatum*. (F) Liquid chromatogram of dendrobine in SG-multigene transgenic *D. catenatum*. (G) Qualitative and quantitative analysis of dendrobine by LC/MS in SG-multigene transgenic *D. catenatum*. This data corresponds to Fig. 1E.

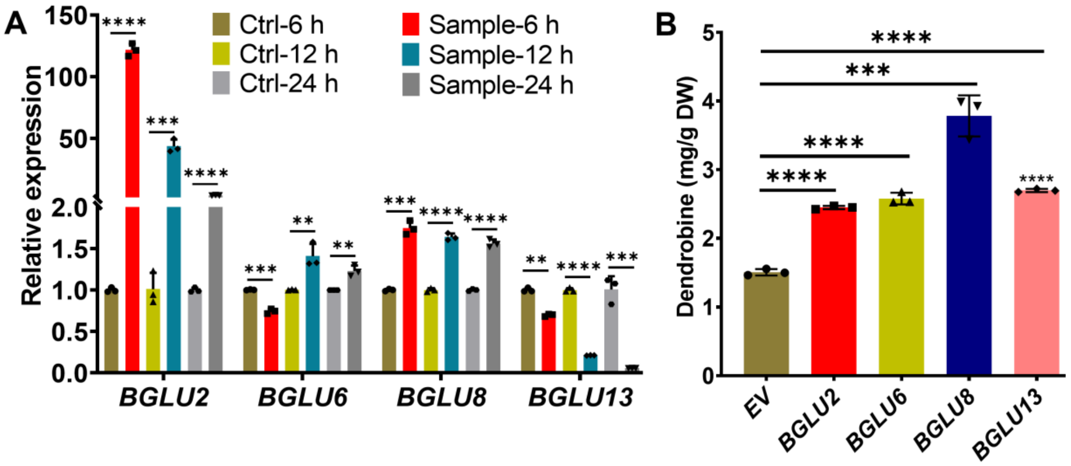

Figure S2. *BGLU*s are regulators of dendrobine synthesis. (A) Gene expression levels of *BGLU2*, *BGLU6*, *BGLU8*, and *BGLU13* in leaves of *D. catenatum* (38-month-old) overexpressing each gene, measured 24 hours after infiltration. (B) Quantification of dendrobine content in the leaves of *D. catenatum* transiently overexpressing *BGLU2*, *BGLU6*, *BGLU8*, and *BGLU13*, conducted by HPLC five days after infiltration.

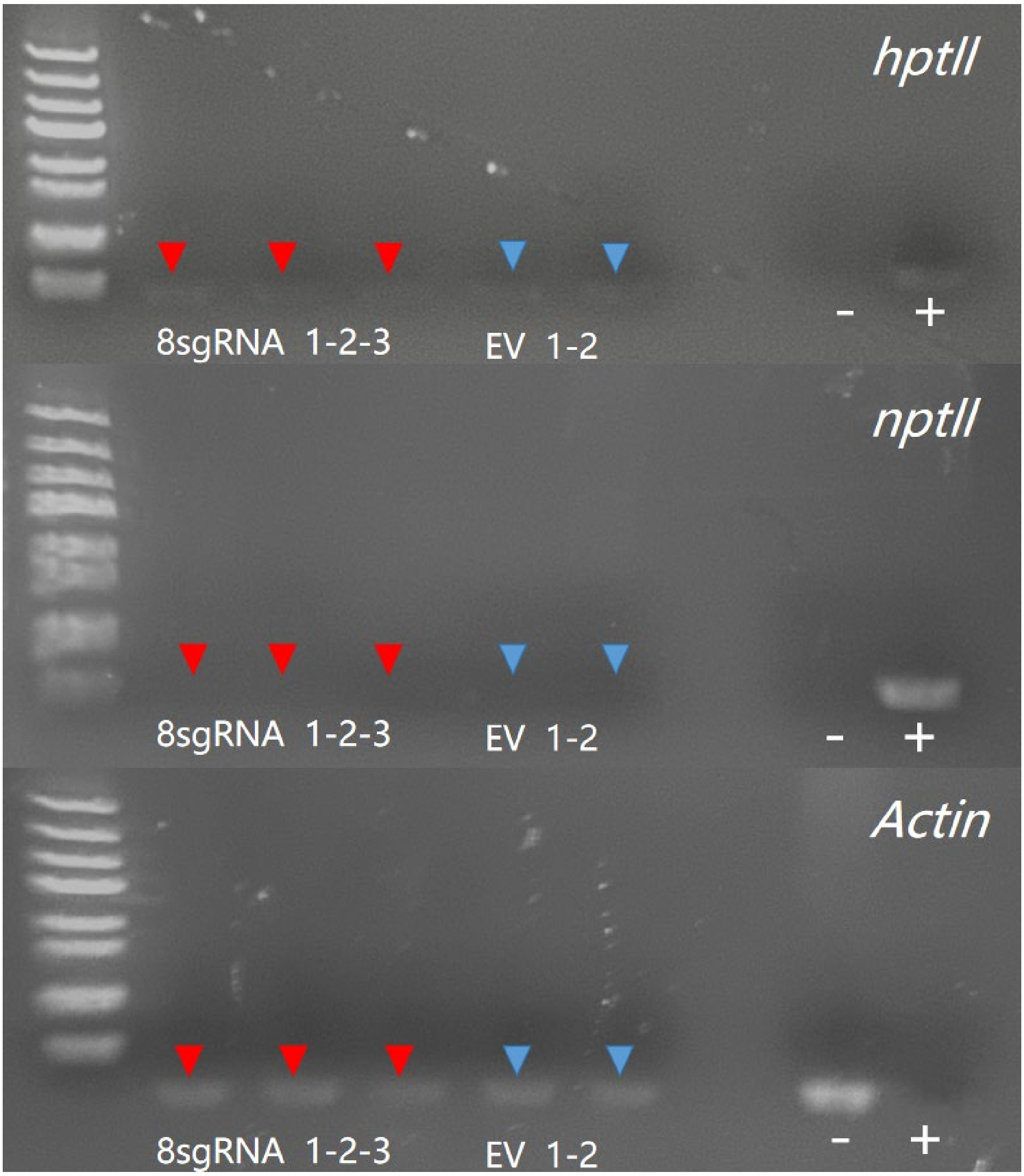

Figure S3. Molecular characterization of the *hptII* transgene in *D. catenatum* transgenic plants. This data corresponds to Figure 4G.

Table S1. Primers used for genomic PCR.

Genes	Name	Sequences
HPTII	Forward	5'-GTTGGCGACCTCGTATTGG-3'
	Reverse	5'-TGACATTGGGGAGTTTAGCG-3'
NPTII	Forward	5'-TGTTGCTGTCTCCCAGGTCG-3'
	Reverse	5'-ACAGCCGCTTAGCCGAAT-3'
ACTIN	Forward	5'-GAAGCCCAGTCCAAAAGAGGTATCC-3'
	Reverse	5'-ACATGGCAGGCACATTGAAAGTCTC-3'

Table S2. sgRNA sequences for CRISPR/Act3.0.

sgRNA	Sequence
DnMCT	5'-CCGGATCCAGGGTAGATGAG-3'
DnHMGR	5'-CCTGATCCTTTCGCTGTGAG-3'
DnSTR1	5'-AAAATTTTAATTGACGGCTA-3'
DnCYP94C1	5'-TTCATTATATAGGAGTTCTG-3'
DnBGLU2	5'-GTTCGTCTGAGCATTCCAGA-3'
DnBGLU8	5'-TGGTCTCGCCAGTGAAAGCG-3'
DnMYB61	5'-ACTTGCCTGTGGGATTGTGG-3'
DnCMEAO	5'-AAAAGGGAAAGAAGGCAACG-3'
DcMCT-1	5'-GATGCAAGAAAGAAAACCTA-3'
DcMCT-2	5'-TCTACGCGGGATCCGGCCTT-3'
DcMCT-3	5'-CGTACTCCGCTCATCTACGC-3'

DcMCT-4	5'-GTAGTTAGATTCCTTTCTGA-3'
DcMCT-5	5'-ACTACAAAGTTCGGTTGAGA-3'
DcHMGR-1	5'-ACCGCGGAGCAAGAGAAAAG-3'
DcHMGR-2	5'-ATCAGGAGGCGGGTGTGTAG-3'
DcSTR1-1	5'-GCTTTTCCCGCGAATACCTT-3'
DcSTR1-2	5'-AAAGCCGCCAAAGGTATTCG-3'
DcCYP94C1-1	5'-GGGCGGCTCTGGGCAATGAT-3'
DcCYP94C1-2	5'-CGGCTCTGGGCAATGATGGG-3'

Table S3. Primers used for qRT-PCR.

Genes	Name	Sequences
DnMCT	Forward	5'-CTGAAGGATGGGTGGCTGAA-3'
	Reverse	5'-AAACCAGCTCTGAGTAAATCGGA-3'
DnHMGR	Forward	5'-CTGGAGATGCAATGGGGATG-3'
	Reverse	5'-CACTTCCTCTTTGATGACTGCCT-3'
DnSTR1	Forward	5'-TATTTTGGGTTGCTGAAGGTAGG-3'
	Reverse	5'-CCTTGTCACCTTTCAGCCAGTAT-3'
DnCYP94C1	Forward	5'-GAGATGGCTCCAAGACGGC-3'
	Reverse	5'-CTTAAGCTCAACCTGTCGGACTC-3'
DnBGLU2	Forward	5'-AGTGGCAGCCAAAAGAGCT-3'
	Reverse	5'-GGGAGCCTTGAACCTGCA-3'
DnBGLU8	Forward	5'-TCCCATCTGGTCCTGCGA-3'
	Reverse	5'-GGACCCTCGCTTGCTGTT-3'
DnMYB61	Forward	5'-GGGAAGGCACTCCTGCTG-3'
	Reverse	5'-GCTTGGGGACAGAGCTCC-3'
DnCMEAO	Forward	5'-CGCCATTCAGGGAAGCCA-3'
	Reverse	5'-CCAATCTGCGGCTAGGGG-3'
ACTIN	Forward	5'-GAAGCCCAGTCCAAAAGAGGTATCC-3'
	Reverse	5'-ACATGGCAGGCACATTGAAAGTCTC-3'
